# Acute brain swelling due to intraoperative supratentorial subdural hematoma during microvascular decompression: a case report

**DOI:** 10.3389/fsurg.2025.1643743

**Published:** 2025-10-24

**Authors:** Jia Shi, Jiachao Cao, Qiang Zhou

**Affiliations:** Department of Neurosurgery, The Third Affiliated Hospital of Soochow University, Changzhou, China

**Keywords:** microvascular decompression, trigeminal neuralgia, subdural hematoma, encephalocele - complications, complication

## Abstract

**Background:**

Microvascular decompression (MVD) is the primary treatment for cranial neurovascular compression syndromes. The most critical complication is hemorrhage during or after surgery. Although intraoperative supratentorial subdural hematoma (SDH) is uncommon, its management remains complex and lacks standardization.

**Case presentation:**

We report a case of acute brain swelling caused by an intraoperative supratentorial subdural hematoma during microvascular decompression (MVD) for trigeminal neuralgia. By effectively managing intracranial pressure and preventing secondary brain injury, the operation was successfully completed, resulting in a favorable postoperative recovery for the patient.

**Conclusion:**

This study examines the presentation and management of acute brain swelling caused by intraoperative supratentorial subdural hematoma (SDH). We aim to highlight the importance of being vigilant for this rare complication during microvascular decompression (MVD), as it can result in fatal brain swelling and encephalocele. Preventing and managing this severe complication remains challenging.

## Background

Microvascular decompression (MVD) is the primary treatment for cranial neurovascular compression syndromes, such as trigeminal neuralgia (TN), hemifacial spasm (HFS), and glossopharyngeal neuralgia (GN). The surgical safety for conditions such as intracranial hemorrhage and infection has been verified. Even advanced age is no longer an absolute contraindication for surgery (1). Although MVD is associated with low morbidity and mortality, severe complications can arise. The most life-threatening of these is intraoperative and postoperative hemorrhage (2–6). Most postoperative intracranial hemorrhages occur infratentorially, with remote hemorrhages being rare (2, 4, 6–8). Intraoperative supratentorial subdural hematomas (SDH) are even less common. A recent report detailed three cases of supratentorial SDH during MVD (9). Furthermore, Wang et al. reported two cases of SDH that occurred during endoscopic MVD in patients with hemifacial spasm, and they hypothesized that this could be linked to excessive cerebrospinal fluid drainage and positioning during the procedure, though no objective evidence supports this claim (10). A retrospective study found that the biological glue suspension method is more effective in relieving postoperative symptoms compared to traditional decompression techniques, despite an increased risk of postoperative hemorrhage (11). We describe a case of acute brain swelling caused by intraoperative supratentorial SDH during MVD for trigeminal neuralgia, a scenario not previously documented in the literature.

## Case presentation

**Figure 1:**
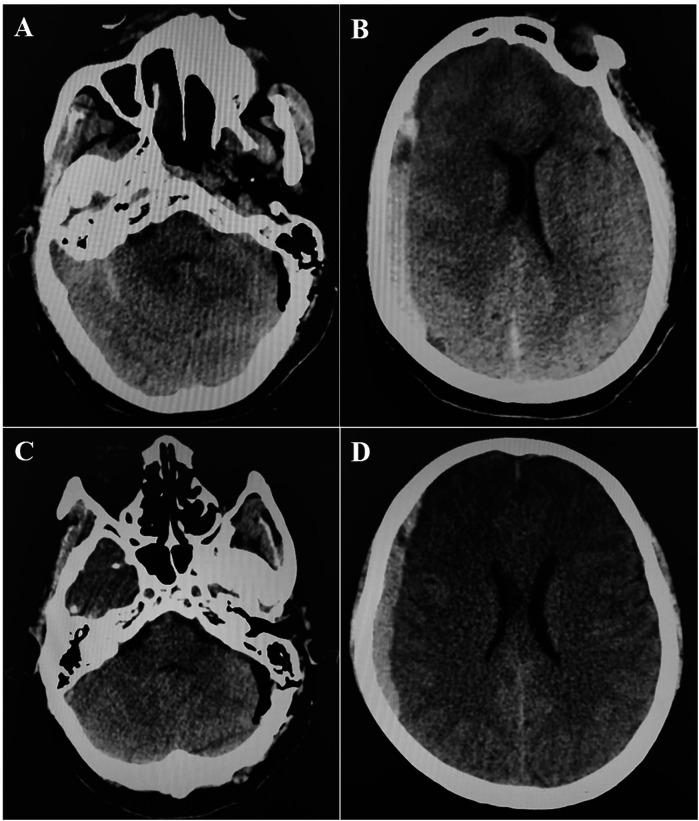
Postoperative CT scan. **A,B**: CT at 0 h post-operation showed that there was no hemorrhage in the operation area of left cerebellopontine angle, but there was right supratentorial subdural hematoma (1 cm thickness). **C,D**: CT at 4 h post-operation showed that hematoma was stable.

**Figure 2:**
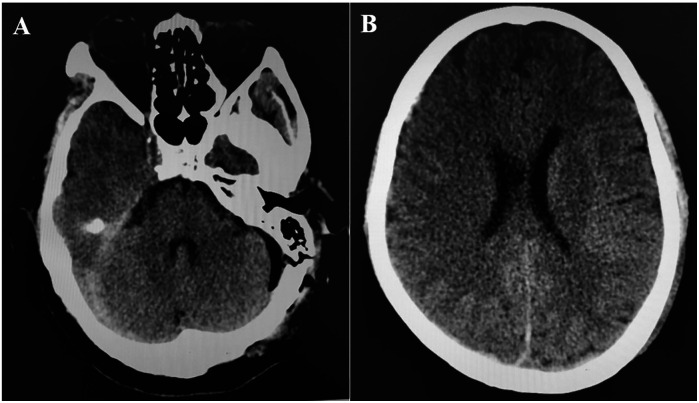
**(A,B)** CT at 21 h post-operation showed that the right subdural hematoma was significantly dissipated.

A retrospective review of 480 patients who underwent MVD for hemifacial spasm, trigeminal neuralgia, or glossopharyngeal neuralgia at our hospital from February 2008 to March 2019 identified one instance of acute brain swelling due to supratentorial subdural hematoma. This case involved a 60-year-old woman with a five-year history of trigeminal neuralgia and no hypertension, coagulopathy, or systemic diseases. During surgery, she was positioned laterally, and the arachnoid membrane was routinely opened. Cerebrospinal fluid (CSF) drained slowly, causing slight cerebellar collapse. Ten minutes after dura entry, the cerebellopontine cistern was opened, leading to a sudden CSF gush and almost immediate acute brain swelling, filling the surgical field within 10 s (Supplementary Video S1). Throughout, the patient's head position remained unchanged, blood pressure and heart rate were stable, anesthesia was maintained, and the procedure followed standard protocols without any unusual circumstances. The cause of the brain swelling was unclear, given normal manipulation, unobstructed airway, and absence of surgical bleeding. We had to manage the brain swelling while considering the possibility of remote hemorrhage. Mannitol (250 ml ivgtt) and dexamethasone (10 mg i.v.) were administered to lower intracranial pressure (ICP), while cerebrospinal fluid was gradually drained from the compact subdural space. The surgical field was restored within 40 min, and ICP gradually decreased. Despite the surgical field being narrower than initially, and elevated ICP detected under the suction device, we proceeded with supracerebellar artery decompression of the trigeminal nerve in a confined space (Supplementary Video S2). Bilateral pupil monitoring during surgery showed normal results. A postoperative CT scan immediately revealed contralateral acute supratentorial subdural hematoma (SDH) with a midline shift of approximately 4 mm (Figure 1). The patient awoke from anesthesia with a Glasgow score of 15, and no obvious signs of neurological dysfunction were observed. Moreover, within 8 h after the operation, three head CT scans were completed, confirming that the volume of the subdural hematoma remained stable and did not increase further. At the same time, the patient's clinical symptoms were stable, with no obvious symptoms of increased intracranial pressure such as headache or projectile vomiting. Therefore, we did not place an ICP probe for continuous monitoring of intracranial pressure, but continued to observe the clinical symptoms and signs. Fortunately, when the CT was rechecked 21 h after the operation, it was found that most of the subdural hematoma had dissipated (Figures 2A,B). The patient was discharged eight days later without neurological impairments.

## Discussion

Postoperative intracranial hemorrhage remains the most common complication of posterior fossa surgery, including microvascular decompression, leading to severe consequences (12, 13). Supratentorial hemorrhage following MVD is relatively uncommon and there are only a few reports available (Supplementary Table S1). Recent literature has documented four cases of supratentorial subdural hematoma (SDH) after MVD (6) and three cases of supratentorial SDH during MVD (9). Several reports have described the clinical details of the complications (2, 6, 9). The three cases of intraoperative SDH all revealed bleeding of unknown origin within the surgical field (9). In the case of Amagasaki et al, all three HFS patients experienced bleeding in the surgical area (or continuous bloody cerebrospinal fluid) during the operation. These symptoms suggested to the surgeons that there was bleeding in the brain. In our case of TN, no bloody cerebrospinal fluid or bleeding in the surgical area was observed, but there was a transient cerebrospinal fluid overflow, which caused confusion in our judgment during the operation. Moreover, acute brain swelling followed immediately in our case, which brought greater difficulties to the surgery. In the case of Amagasaki's team, one patient underwent a second operation (to remove the subdural hematoma) and suffered from the sequelae of Gerstmann's syndrome and epilepsy. Fortunately, in this case, the subdural hematoma of the patient quickly dissipated and the neurological function was intact (9). Literature primarily discusses intraoperative bloody cerebrospinal fluid or operative site bleeding, as well as remote subdural hematomas identified during routine postoperative evaluations. In our case, no operative site bleeding was observed; however, significant brain swelling occurred, following a brief cerebrospinal fluid outflow. We propose that transient cerebrospinal fluid efflux and unexplained intraoperative brain swelling may indicate subdural hematoma at remote locations, warranting attention. The choice of hemostatic method during surgery depends on the surgical phase and the specific anatomical site of bleeding. Surgeons must be familiar with various hemostatic techniques to effectively manage bleeding, particularly in complex cranial neurosurgical cases (14). Novel hemostatic agents are utilized to manage intraoperative bleeding. The use of autologous fibrin sealant has been effective in controlling cerebral bleeding and promptly sealing the dura, leading to the resolution of cerebrospinal fluid leaks (15). IEIK13 demonstrates efficacy and safety in managing oozing hemorrhage in intracranial neurosurgical procedures. This trial supports the utility of the transparent IEIK13 hydrogel as a valuable tool for achieving hemostasis in neurosurgery (16). Among our 480 patients who underwent microvascular decompression for hemifacial spasm, trigeminal neuralgia, and glossopharyngeal neuralgia, three developed SDH. These hematomas were minor and caused no neurological deficits. While acute brain swelling from intraoperative supratentorial SDH during MVD is exceedingly rare and perilous, we found no comparable cases in the literature.

**Figure 3:**
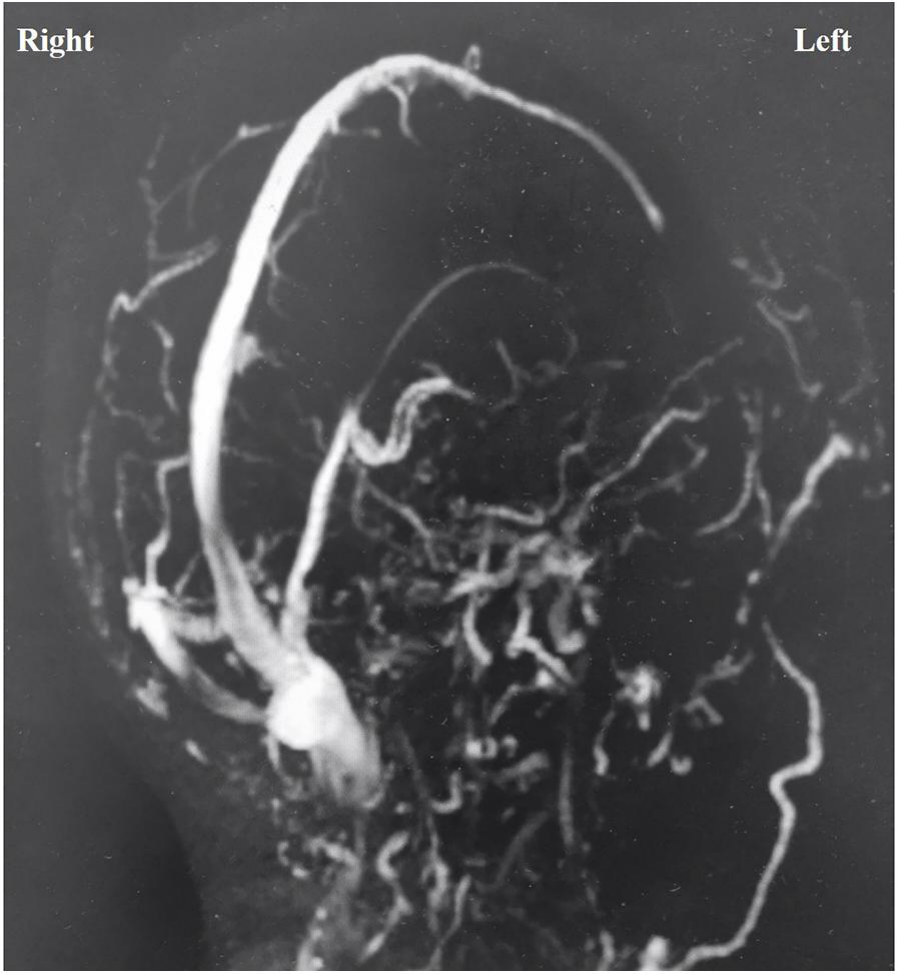
Magnetic resonance venography showed that the left transverse sinus and sigmoid sinus were not visualized, and the venous drainage was mainly on the right side.

The cause of supratentorial subdural hematoma (SDH) during posterior fossa surgery remains uncertain. However, potential mechanisms include excessive cerebrospinal fluid (CSF) drainage and neck rotation and flexion in the lateral decubitus position (2, 4). Nozaki reported that transitioning patients from the intraoperative lateral position to the postoperative supine position resulted in low intracranial pressure and/or intracranial air, causing the brain to shift and subsequently leading to supratentorial subdural hematoma (6). This hypothesis fails to account for intraoperative bleeding. Recent studies indicate that most subdural hematomas occur on the nondominant side of venous drainage; however, no definitive link exists between venous drainage laterality and bleeding (6, 9). In our case, both the rate and volume of CSF drainage were normal, as previously noted. However, CSF unexpectedly surged from the cistern, which we attributed to increased ICP from the supratentorial SDH, rather than as a cause of it. The hematoma was located on the dominant side of venous drainage (Figure 3), but the relationship between supratentorial SDH and venous pressure remains unclear. Avoiding this rare complication is nearly impossible due to its unknown etiology, and a sudden intraoperative rise in CSF outflow might indicate its occurrence.

Acute brain edema is commonly seen in superior cerebral edema after trauma or obstructive hydrocephalus, leading to trans-tentorial brain hernia and requiring urgent treatment. Secondary direct temporal lobe disengagement in traumatic brain injury may offer an approach to managing trans-tentorial brain herniation, potentially avoiding more complex interventions. Encouraging clinical outcomes suggest that this method could positively impact patients' neurological recovery (17). Otherwise, Cerebellum edema, along with obstructive hydrocephalus, is an uncommon manifestation of hypertensive encephalopathy. The authors describe a unique instance of isolated swelling in the posterior fossa, leading to upward trans-tentorial herniation and subsequent hydrocephalus, resulting in neurological decline (18). In such instances, intraoperative ultrasound provides real-time imaging of structures within the surgical field, aiding surgeons in aligning intraoperative anatomical images. Intraoperative ultrasound proves to be a versatile tool in surgery, as it aids in identifying CSF and blood flow, assessing tissue characteristics, detecting peripheral infiltration, and promptly visualizing complications such as hematomas (19). Residual bleeding or hematoma during the operation is an important cause of brain edema. Visible near-infrared spectroscopic (VNIRS) is a non-invasive real-time intraoperative monitoring technology that has been applied in neurosurgery (20). It provides a reliable and convenient way for surgeons to detect and locate hematomas during the operation (21).

Acute brain swelling due to intraoperative supratentorial SDH presents a complex and hazardous challenge, necessitating urgent intervention. Determining the cause and severity of this swelling remains uncertain for surgeons, complicating decision-making. Despite the lack of a standardized approach, certain principles are essential. First, controlling brain swelling is crucial to prevent acute encephalocele, which can lead to severe, irreversible outcomes. The most effective strategy is the gradual release of CSF, requiring patience and precision to avoid brain tissue damage. Second, if abnormal pupils are detected or imaging reveals a significant remote hematoma, immediate skull closure and rapid hematoma evacuation may be imperative. In this case, the surgeon successfully managed the brain swelling, allowing for the completion of the MVD, and the hematoma resolved spontaneously by the second postoperative day.

## Conclusion

Learning Points: Vigilance during sudden CSF gush, gradual drainage, importance of immediate imaging.

Surgeons must remain vigilant for intraoperative supratentorial SDH during MVD, despite its rarity, as it can result in fatal acute brain swelling and encephalocele. Preventing and managing this severe complication remains challenging.

## Data Availability

The original contributions presented in the study are included in the article/Supplementary Material, further inquiries can be directed to the corresponding author.
