# Supplementary material for: Acute brain swelling due to intraoperative supratentorial subdural hematoma during microvascular decompression: a case report
Source: Front Surg. 2025 Oct 24;12:1643743. doi: 10.3389/fsurg.2025.1643743 (PMC12592028; doi:10.3389/fsurg.2025.1643743)
Supplement: Supplementary file 1 [file Datasheet1.pdf]

Supplement Table 1. Summary of four previously reported SDH in MVD

Author	SDH Timing	Management	Outcome
Amagasaki et al[1].	Intraoperation	Removal of the heamatoma (case 1); Concervative treatment (case 2/3)	Gerstmann's syndrome and epilepsy (case 1); no deficit (case 2/3)
Nozaki et al[2].	Postoperation	Concervative treatment	No deficit
Lee et al[3].	Postoperation	Concervative treatment	No deficit
Jing et al[4].	Intraoperation	ICP (case 1); concervative treatment (case 2)	No deficit

ICP: intracranial pressure.

Reference

1. Amagasaki K, Takusagawa Y, Kanehashi K, et al. Supratentorial acute subdural haematoma during microvascular decompression surgery: report of three cases. J Surg Case Rep 2017;2017(2):rjx004. (In eng). DOI: 10.1093/jscr/rjx004.
2. Nozaki T, Sugiyama K, Sameshima T, Kawaji H, Namba H. Supratentorial subdural hematoma following microvascular decompression: a report of four cases. Springerplus 2016;5:353. (In eng). DOI: 10.1186/s40064-016-2002-2.
3. Lee MH, Jee TK, Lee JA, Park K. Postoperative complications of microvascular decompression for hemifacial spasm: lessons from experience of 2040 cases. Neurosurg Rev 2016;39(1):151-8; discussion 158. (In eng). DOI: 10.1007/s10143-015-0666-7.
4. Wang J, Wang X, Luo T, Wang X, Qu Y. Supratentorial Acute Subdural Hematoma During Fully Endoscopic Microvascular Decompression Surgery for Hemifacial Spasm. J Craniofac Surg 2023;34(2):e187-e190. (In eng). DOI: 10.1097/scs.0000000000008987.
